# Potent and Broad-Spectrum Antimicrobial Activity of Analogs from the Scorpion Peptide Stigmurin

**DOI:** 10.3390/ijms20030623

**Published:** 2019-01-31

**Authors:** Bruno Amorim-Carmo, Alessandra Daniele-Silva, Adriana M. S. Parente, Allanny A. Furtado, Eneas Carvalho, Johny W. F. Oliveira, Elizabeth C. G. Santos, Marcelo S. Silva, Sérgio R. B. Silva, Arnóbio A. Silva-Júnior, Norberto K. Monteiro, Matheus F. Fernandes-Pedrosa

**Affiliations:** 1 Laboratory of Pharmaceutical Technology and Biotechnology, Pharmacy Department, Federal University of Rio Grande do Norte, Natal, Rio Grande do Norte 59012-570, Brazil; bruno_portilo@hotmail.com (B.A.-C.); alessandra.daniele@outlook.com (A.D.-S.); adrianamsparente@gmail.com (A.M.S.P.); allannyfurtado@hotmail.com (A.A.F.); elizabethcgsantos@gmail.com (E.C.G.S.); arnobiosilva@gmail.com (A.A.S.-J.); 2 Laboratory of Bacteriology, Instituto Butantan, São Paulo 05503-900, Brazil; eneas.carvalho@butantan.gov.br; 3 Immunoparasitology Laboratory, Department of Clinical and Toxicological Analysis, Federal University of Rio Grande do Norte, Natal 59012-570, Brazil; johny3355@hotmail.com (J.W.F.O.); mssilva.ufrn@gmail.com (M.S.S.); 4 Global Health and Tropical Medicine, Institute of Hygiene and Tropical Medicine, Universidade Nova de Lisboa, 1099-085 Lisbon, Portugal; 5 Brain Institute, Federal University of Rio Grande do Norte, Natal, Rio Grande do Norte 59056-340, Brazil; sergioruschi@neuro.ufrn.br; 6 Analytical Chemistry and Chemical Physics Department, Federal University of Ceará, Fortaleza, Ceará 60455-900, Brazil; norbertokv@ufc.br

**Keywords:** *Tityus stigmurus*, analog peptides, scorpion venom, antimicrobial agent, molecular dynamics, bacterial membrane

## Abstract

Scorpion venom constitutes a rich source of biologically active compounds with high potential for therapeutic and biotechnological applications that can be used as prototypes for the design of new drugs. The aim of this study was to characterize the structural conformation, evaluate the antimicrobial activity, and gain insight into the possible action mechanism underlying it, for two new analog peptides of the scorpion peptide Stigmurin, named StigA25 and StigA31. The amino acid substitutions in the native sequence for lysine residues resulted in peptides with higher positive net charge and hydrophobicity, with an increase in the theoretical helical content. StigA25 and StigA31 showed the capacity to modify their structural conformation according to the environment, and were stable to pH and temperature variation—results similar to the native peptide. Both analog peptides demonstrated broad-spectrum antimicrobial activity in vitro, showing an effect superior to that of the native peptide, being non-hemolytic at the biologically active concentrations. Therefore, this study demonstrates the therapeutic potential of the analog peptides from Stigmurin and the promising approach of rational drug design based on scorpion venom peptide to obtain new anti-infective agents.

## 1. Introduction

Antimicrobial resistance is a severe global health problem and has become one of the main causes of morbidity and mortality in the world. The high spontaneous mutation rate and genetic recombination of the microorganisms, associated with the irrational use of antibiotics, have promoted the prompt development of multi-resistant pathogens, leading to research on new antimicrobial molecules from different natural sources [1].

Scorpion venom constitutes a complex mixture of molecules with high therapeutic potential [2]. Venom peptides with insecticide, antiviral, antimicrobial, hemolytic, antiproliferative, bradykinin potentialization, and immunomodulatory activities, which have been described in previous studies, are targeted in the prospection of novel molecules of biotechnological interest [3]. Antimicrobial peptides (AMPs) without disulfide bridges have been identified in different scorpion species, presenting a positive net charge and a hydrophobic character, which permit interaction with microorganism cellular membranes [4]. AMPs usually present random coil conformation in polar solvent, but show an α-helical conformation when they interact with membranes or non-polar solvents [5]. The mechanism of action of the AMPs can be described as follows: First, the electrostatic interaction occurs between its positive amino acids with the negatively charged phospholipids in the microorganism membrane; then a displacement of lipids is induced, leading to pore formation [6]. Accordingly, the bacterial sensitivity is directly related to the different composition and physicochemical properties of the lipids in cellular membranes [7].

In eukaryotic membranes, lipids in the outer membrane are commonly neutral phospholipids, such as phosphatidylserine and sphingomyelin. However, the bacterial cellular membrane is mainly composed of negatively charged lipids, such as phosphatidylglycerol (PG), cardiolipin, and phosphatidylethanolamine (PE) [8]. The negative net charge in bacterial membranes plays an important role in the preferential interaction of cationic AMPs and microorganism membranes [1].

Several structural determinants are responsible for the antimicrobial and cytolytic activity of antimicrobial peptides. Studies have shown that increased α-helical conformation, cationic character, hydrophobicity and hydrophobic moment of native antimicrobial peptides have an effect on the spectrum of antibiotic action. [9,10,11]. Another important factor for the use of these molecules is their amidation in the C-terminal region, as it has demonstrated a greater protection to the proteolysis and a greater structural stability of the amphipathic helix [12]. However, a clearer understanding of the influence of these characteristics on the peptide structure may help to develop new strategies that will facilitate the creation of new pharmacological agents that improve or optimize mechanisms to suppress the capacity of pathogens to generate resistance, as well as to improve the potential therapeutic use of these peptides. 

Stigmurin is an AMP without disulfide bridges with 17 amino acid residues, obtained from the transcriptome analysis of the scorpion *Tityus stigmurus* venom gland by our research group. This antimicrobial peptide presented in vitro and in vivo antimicrobial activity and low hemolytic activity [13,14]. Amino acid substitutions in a short peptide chain have been reported to improve the native peptide characteristics, increasing its biological activity and reducing its toxicity. This is a promising approach to develop new molecules with therapeutic potential [15]. To design the analogs of this study, we replaced the polar and uncharged Ser and Gly residues of the native peptide with positively charged Lys residues in order to increase the positive charge of Stigmurin and observe the effect of increasing the charge on antimicrobial activity and hemolytic activity of this non-hemolytic peptide with a low positive charge (+2). Moreover, the structural conformation in silico of the two analog peptides, denominated as StigA25 and StigA31, was assessed by circular dichroism, as well as their antimicrobial activity in vitro and in silico by molecular dynamics.

## 2. Results

### 2.1. In Silico Analysis 

Stigmurin and the analog peptides presented predominantly α-helical structures in silico. An increase in α-helix percentage and hydrophobic moment was observed with the amino acid substitutions for lysine (K) (Table 1).

### 2.2. Molecular Models

**Figure 1:**
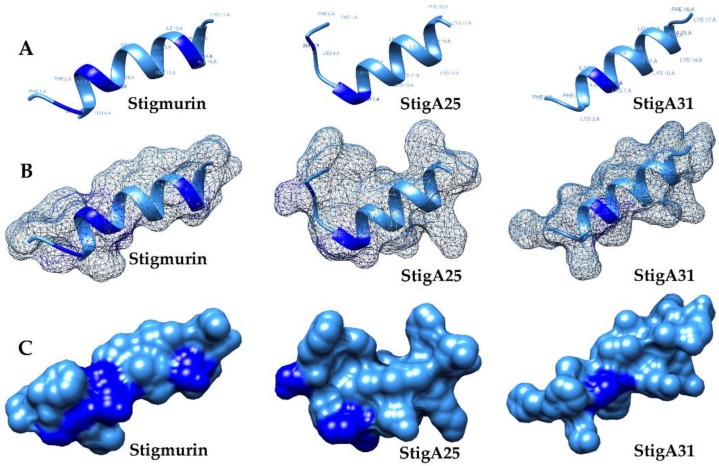
Theoretical tridimensional structure by AIDA using UCSF Chimera software for the peptides Stigmurin, StigA25, and StigA31. In (**A**) peptides in surface model, (**B**) mesh model, and (**C**) electrostatic surface. Light blue and dark blue represent hydrophobic and hydrophilic regions, respectively.

The Molprobity scores were 1.78, 1.33, and 0.50 for Stigmurin, StigA25, and StigA31, respectively. The generated theoretical models presented 86%, 98%, and 100% of amino acids in favored regions for Stigmurin, StigA25, and StigA31, respectively, indicating that quality models were predicted. The models showed a predominant helical structure (Figure 1A,B). Moreover, an amphipathic surface can be observed for Stigmurin, StigA25, and StigA31 (Figure 1C), indicating that these peptides can potentially interact with anionic membranes.

### 2.3. Molecular Dynamics (MD)

**Figure 2:**
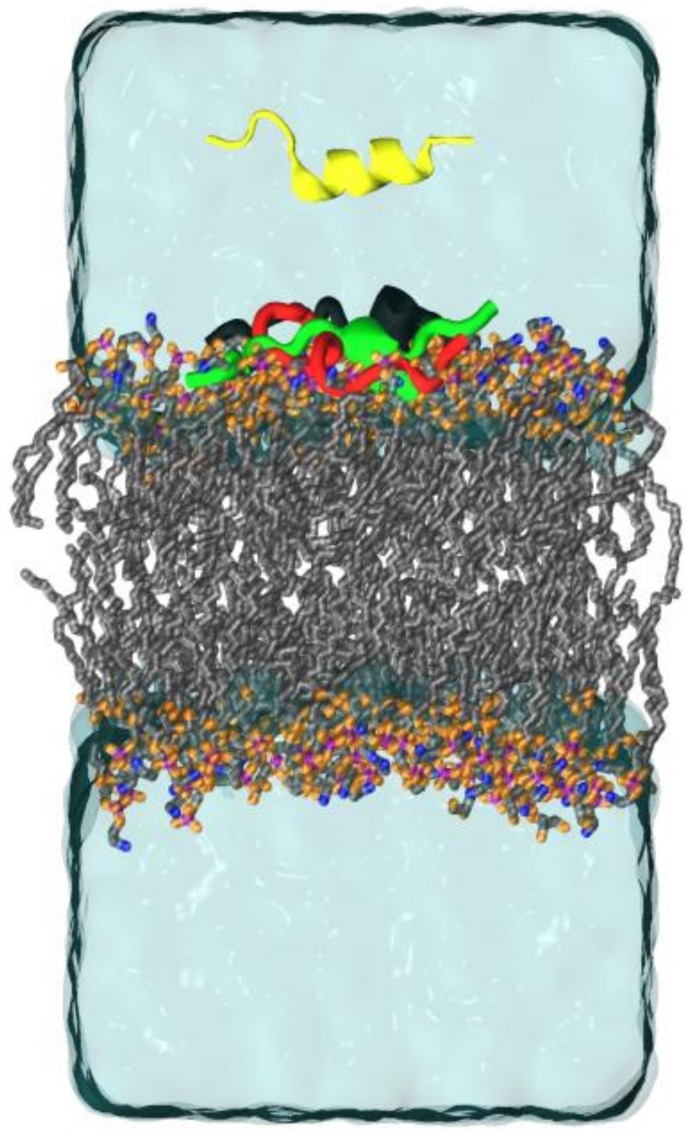
Schematic view of system submitted to MD simulations of Gram-negative bacteria mimicked. XZ plane representation of system with peptide (yellow) above lipid bilayer representing initial condition for all peptides simulated and Stigmurin (black), StigA25 (red) and StigA31 (green) presenting their respective final condition. The lipid molecules are represented as lines, with carbon atoms in gray, nitrogen in blue, oxygen in orange, and phosphorus in purple.

**Figure 3:**
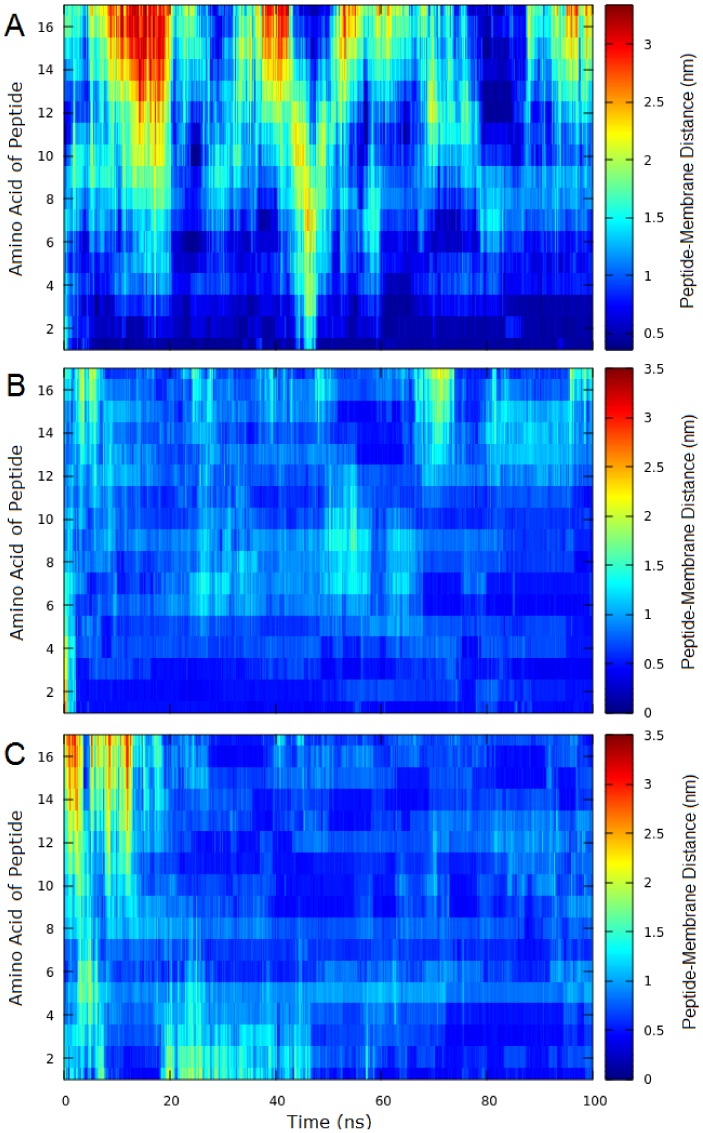
Peptide–membrane distance over time by amino acid during 100 ns MD simulations for (**A**) Stigmurin, (**B**) StigA25 and (**C**) StigA31. The distance between the atoms (except hydrogen) of each amino acid of the peptides and phosphorus atom (hydrophilic head of the lipids) was considered.

A total time of 100 ns simulations was enough to equilibrate the lipid bilayer membranes. Structural stabilization occurred around 50 ns. In this model for Gram-negative bacteria in absence of peptides, with molar ratio POPG/POPE 1:3, the published values of area per lipid (Å^2^) and membrane thickness (Å) was found to be 58.34 ± 1.33 and 41.65 ± 0.78, respectively [16]. The values obtained from our MD simulations were 57.7 ± 0.7 and 42.17 ± 0.3 for same parameters. The same time was applied for system with isolated peptides. The root mean square deviation (RMSD) of the Cα-carbons reached a constant value around ∼0.55 nm (Figure A7) also indicating a structural stabilization. Figure 2 shows the initial configuration of system simulated and final geometry obtained for peptides Stigmurin, StigA25, and StigA31 with lipid bilayers at the end of the 100 ns MD simulation. Figure 3 present the dynamics of the peptide approximation (Stigmurin, StigA25 and StigA31) to the lipid bilayer through the distance between each peptide amino acid to the nearest phosphorous (hydrophilic head of lipids) atom in membrane. We can see from Figure 3 that the peptides presented different approximation behaviors to lipid bilayer throughout the simulation. Stigmurin (Figure 3A) does not approach the membrane evenly while the analogues Stig25 (Figure 3B) and StigA31 (Figure 3C) show larger contact area due to the lower peptide–membrane distance for most of their amino acids during whole simulation. Hence, they have a more negative IPE values of −258.29 (±43.74) kcal mol^−1^ and −313.31 (±36.63) kcal mol^−1^ respectively, compared to Stigmurin’s IPE value of −89.25 (±33.05) kcal mol^−1^. The IPE value of Stigmurin is a direct consequence of the smaller surface area coverage of this peptide with lipid bilayer and, therefore, decreasing the interaction efficiency.

**Figure 4:**
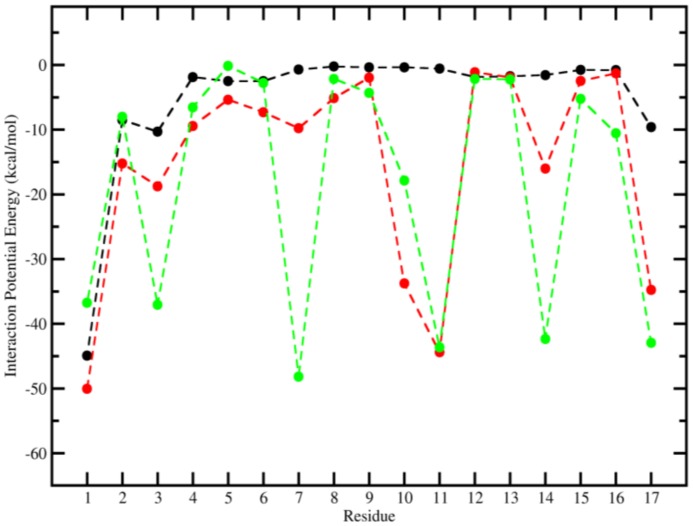
Interaction Potential Energy (kcal mol^−1^) between peptide residues and lipid bilayer over the last 50 ns from MD simulations. Stigmurin (black), StigA25 (red) and StigA31 (green).

**Figure 5:**
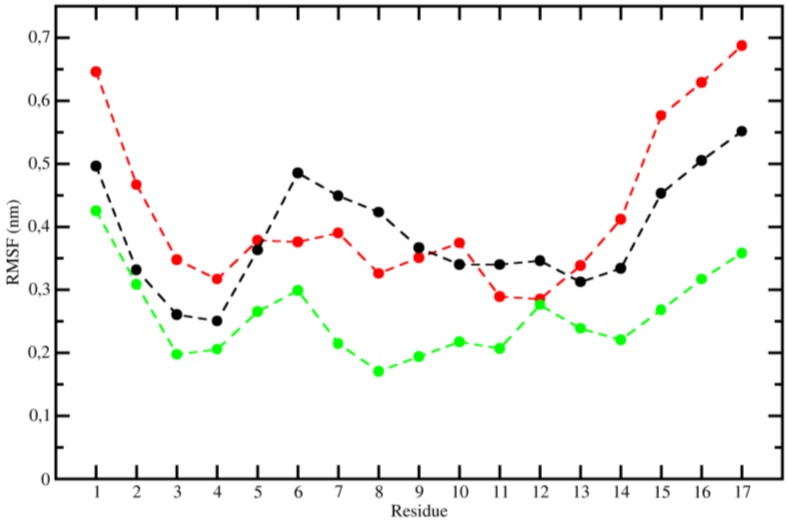
Root mean square of the fluctuation (RMSF) per peptide residues over 100 ns from MD simulations. Stigmurin (black), StigA25 (red), and StigA31 (green).

The analysis of the IPE between lipid bilayer and each individual residue may explain the greater antimicrobial activity of the analogs relative to Stigmurin and provide the reason why the modification in these positions significantly alters the activity observed experimentally. The Figure 4 shows the IPE values between peptides residues and lipid bilayer over last 50 ns from MD simulations. The mutated residues, 10, 11, and 14 (for StigA25) and 3, 7, 10, 11, and 14 (for StigA31) had lower IPE values (around 30 to 40 kcal mol^−1^) when compared to same positions in Stigmurin. The insertion of a positively charged side chain made the existing interaction more stable due to the anionic nature of lipid bilayer. Figure 5 confirms the great fluctuation of terminal residues which move quite well throughout the simulation due to their coil conformation. Because to their high mobility, the residues present higher values of IPE when compared to the more central amino acids of the peptides. This result corroborates that presented in Figure 3 and Figure 4.

### 2.4. Structural Conformation by Circular Dichroism (CD)

**Figure 6:**
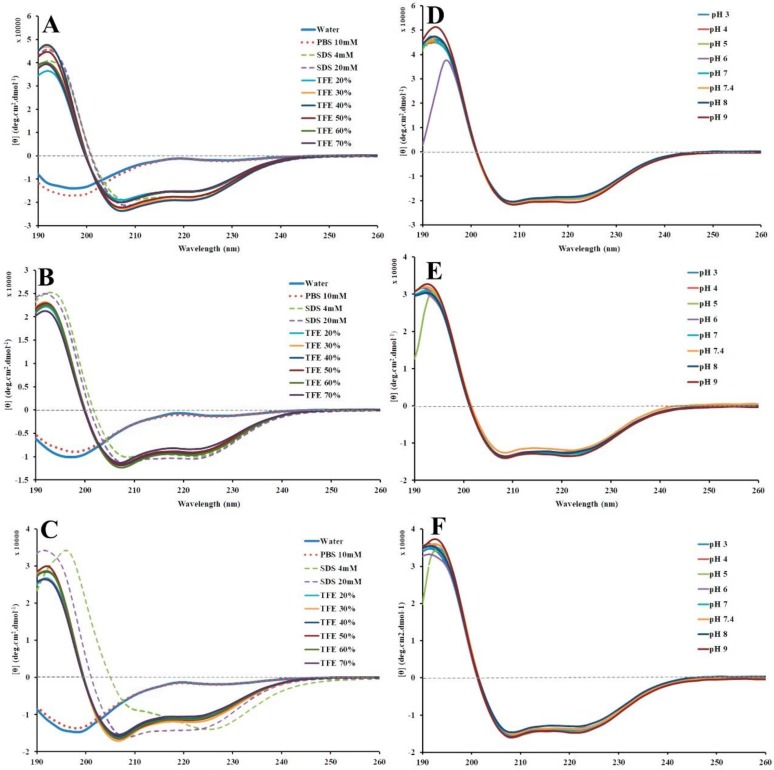
Secondary structure of the peptides evaluated by circular dichroism. Peptides were evaluated in different solvents (Stigmurin—**A**, StigA25—**B**, and StigA31—**C**) and also in different pH (3.0–9.0) in 20 mM SDS (Stigmurin—**D**, StigA25—**E**, and StigA31—**F**). The analyses were performed in a JASCO J-810 spectropololarimeter at 25 °C, with a 1 mm quartz cuvette, from 190 nm to 260 nm at a speed of 50 nm min^−1^.

Stigmurin, StigA25, and StigA31 evaluation using CD in aqueous solvent (water and PBS) demonstrated a typical random coil spectrum (Figure 6A–C). However, when analyzed in hydrophobic solvents, these peptides showed a characteristic α-helix profile, with valleys at 222 nm and 208 nm and a peak at 193 nm [17] (Figure 6A–C). The α-helix secondary structure percentage after deconvolution of CD data was 68%, 41%, and 54% in SDS 20mM for Stigmurin, StigA25, and StigA31, respectively. In the TFE analysis, Stigmurin presented 70% of α-helix structure in TFE 40%, higher than the analog peptides: StigA25 in TFE 60% showed 43% of α-helix and StigA31 in TFE 30% showed 52% of α-helix (Table 2).

### 2.5. Peptide Stability by Circular Dichroism

**Figure 7:**
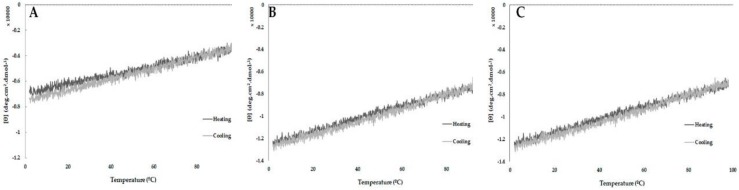
Thermal stability, based on secondary structure evaluation by circular dichroism, of Stigmurin (**A**), StigA25 (**B**), and StigA31 (**C**). CD analyses were carried out in 20 mM SDS with temperatures ranging from 2.0 to 98 °C and then back from 98 to 2.0 °C (1 °C per minute). The ellipticity was recorded every 0.1 °C during heating and cooling at wavelength of 222 nm on a JASCO J-810 spectropolarimeter.

All tested peptides presented high stability to a broad pH range (3.0–9.0) (Figure 6D–F; and Table 3) and temperature (2–98 °C) (Figure 7), where we also observed the peptide capacity to return to its initial conformation after a heating and cooling process.

### 2.6. Hemolytic Activity

**Figure 8:**
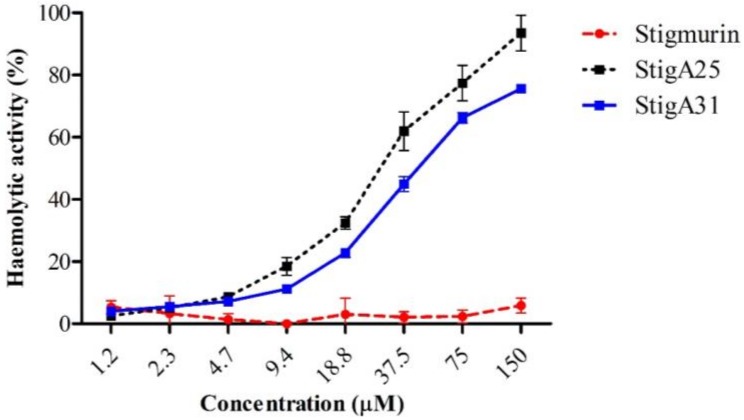
Hemolytic activity percentage of Stigmurin, StigA25, and StigA31.

Stigmurin presented low hemolytic activity (5.8%) at the highest tested concentration (150 µM). We observed that the hemolysis was concentration-dependent for the analog peptides. StigA25 and StigA31 showed low hemolytic activity at concentrations lower than 9.4 µM (18.5% and 11.2%) (Figure 8).

### 2.7. Antimicrobial Activity

StigA25 and StigA31 exhibited potent antimicrobial activity and broad-spectrum action to various medical important strains. The analog peptides StigA25 and StigA31 showed minimum inhibitory concentration (MIC) lower than 4.7 μM and 2.3 μM, respectively, in Gram-positive bacteria. Stigmurin presented MIC of 9.4 μM to *S. aureus* and *S. epidermidis* strains, and was not effective against *Enterococcus faecalis* and Gram-negative bacteria at the concentrations tested. The analog peptides presented high antimicrobial activity in Gram-negative bacteria, with MICs lower than 18.8 and 4.7 μM for StigA25 and StigA31, respectively. Moreover, the analog peptides showed higher activity than Stigmurin for *Candida* yeasts, with higher activity than the standard antibiotics Vancomycin, Gentamicin, and Amphotericin B (Table 4).

### 2.8. Antiparasitic Activity

**Figure 9:**
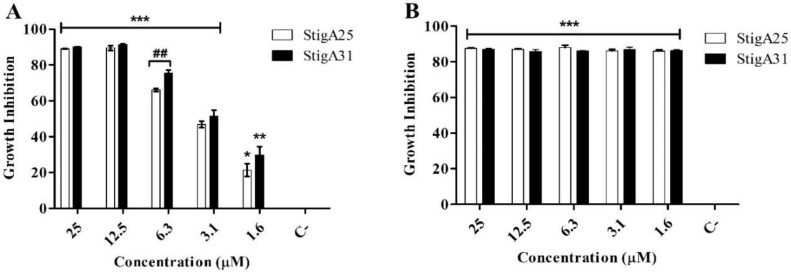
Antiparasitic activity of StigA25 and StigA31 on epimastigote forms of *T. cruzi* after 12 h (**A**) and 24 h (**B**) of incubation. Negative control is represented by C-. Values represent mean ± SD (*N* = 3). *** *p* ≤ 0.0001, ** *p* ≤ 0.001 and * *p* ≤ 0.01 compared to the positive control. ## *p* ≤ 0.001 compared between the analogs StigA25 and StigA31 at the same concentration. Statistical analysis was performed using ANOVA followed by Tukey’s test.

In the antiparasitic assay against the epimastigote forms of *T. cruzi*, the peptides StigA25 and StigA31 (Figure 9) presented antiparasitic activity, inducing 90% of parasite death after 12 h incubation (Figure 9A) at 12.5 μM and 25 μM concentrations. The antiparasitic activity of both analog peptides is similar at 12 h incubation, and were maintained until 24 h incubation (Figure 9B), when we could observe an increase in the parasite death, with 90% inhibition for all concentrations lower than 12.5 μM.

**Figure 10:**
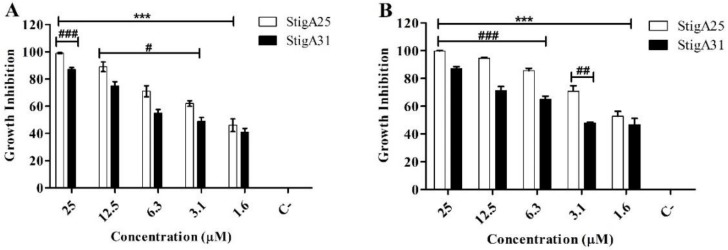
Antiparasitic activity of StigA25 and StigA31 on trypomastigote forms of *T. cruzi* after 12 h (**A**) and 24 h (**B**) of incubation. Negative control is represented by C-. Values represent mean ± SD (*N* = 3). *** *p* ≤ 0.0001 compared to the positive control. ### *p* ≤ 0.0001, ## *p* ≤ 0.001 and # *p* ≤ 0.01 compared between the analogs StigA25 and StigA31 at the same concentration. Statistical analysis was performed using ANOVA followed by Tukey test.

For the trypomastigote forms (Figure 10), an inhibition profile similar to that of the epimastigote forms was observed in 12 h incubation (Figure 10A) for both analogs. However, within 24 h of incubation, the StigA25 peptide showed higher inhibition rates compared to StigA31, with 100% inhibition in 25 μM (Figure 10B).

### 2.9. Mechanism Associated with the Antimicrobial Effect of Peptides in S. aureus

**Figure 11:**
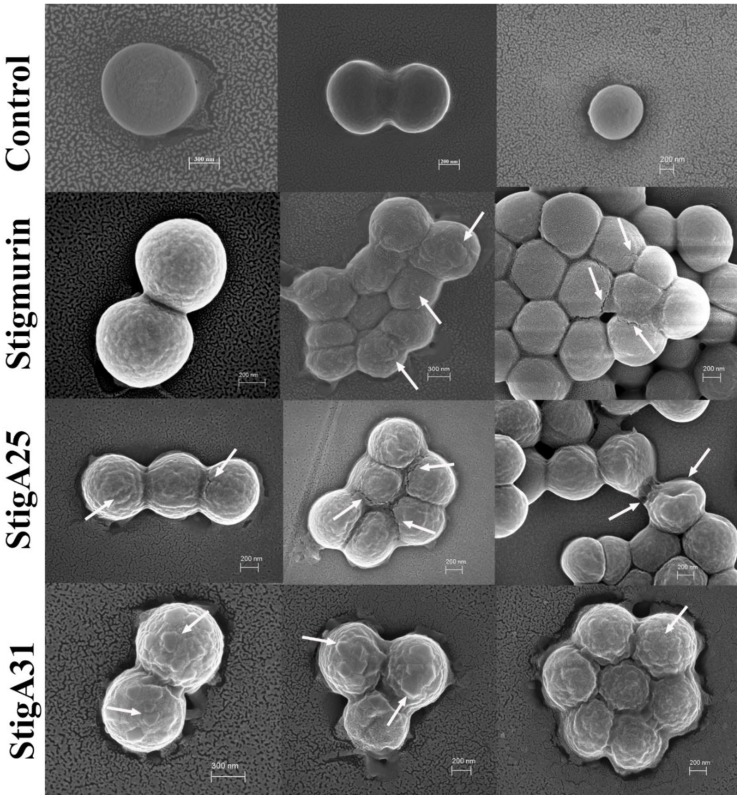
SEM image of *S. aureus* treated with different concentrations of Stigmurin, StigA25 and StigA31 after 18 h. The arrows indicate the changes promoted in the smooth surface of *S. aureus* cells by the peptides, making it possible to observe protuberances and cracks in the cell wall. Three images were used for the control and for each peptide to demonstrate all the morphological changes caused.

It was observed in *S. aureus* cells treated with Stigmurin, StigA25 and StigA31 peptides, several protuberances and cracks on the surface of the cells in MIC concentrations, caused by the interaction and insertion of the peptides in the outer layer of the microorganisms. These changes are more pronounced in the analogs peptides when compared to the native peptide Stigmurin, being more evident in the analogue StigA31, and may be associated with the greater interaction force with the bacterial wall due to the higher liquid surface charge of this peptide. In addition, these results indicate that the outer layer of the *S. aureus* cells were disrupted (Figure 11), without promoting cell lysis or pore formation. Where possibly the synthesis of the peptidoglycan layer has been disrupted and can be observed by displacing the cell wall by cracking but maintaining the shape of the cell.

## 3. Discussion

Antimicrobial peptides (AMPs) are small molecules that act as part of the innate immune system against pathogenic microorganisms [18]. These peptides usually present a general toxic effect on normal cell lines, as well as induce hemolysis in pharmacological concentrations [19]. In this study, the amino acid substitutions on the native peptide Stigmurin for lysine residues (K) resulted in the increase of the α-helix structure, positive net charge, and hydrophobic moment percentages in the analog peptides, as analyzed by in silico methods. The structural models obtained by ab initio modeling for Stigmurin, StigA25, and StigA31 also showed a structure mainly composed of α-helix and hydrophobic residues (approximately 64.7%), with the potential to interact with non-polar compounds [20]. The amino acid substitutions in the native sequence for lysine residues (K) confer a higher cationicity and helical conformation to the analog peptides, and these characteristics have been related to an increase in antimicrobial activity [21]. The molecule amphipathicity is an important factor for the AMP activity in bacteria, which enables the peptide interaction with the lipid bilayer compounds, and this can compromise the bacterial membrane integrity or inhibit essential cellular compounds, reducing the development of resistance. The sum of these characteristics gives these AMPs great importance in microorganism membrane recognition and interaction [20].

In our study, StigA31 (+7) showed a higher negative IPE in the in silico analysis of lipid bilayers when compared with Stigmurin (+2) and StigA25 (+5), indicating a stronger intermolecular interaction. The compilation of these results suggests that StigA31 showed the highest in vitro antimicrobial activity due to the highest IPE value (more negative value) of StigA31 compared to A25 and Stigmurin (−313.31 kcal mol^−1^, −258.29 kcal mol^−1^ and −89.25 kcal mol^−1^, respectively). These results indicate that the mechanism of action of these AMPs is directly associated with electrostatic (Coulomb) interactions that are more dominant than the Van der Waals (Lennard-Jones) interactions. The mutated positions play a crucial role in the antimicrobial efficiency of the peptides due to the insertion of positive charges in the side chains that make the interaction stronger with the anionic membrane. The data reaffirm the predictive capacity of MD simulations and brings the future perspective of studying new analogues through theoretical methods.

The capacity of cationic peptides without disulfide bridges to modify their structural conformation according to the environment provides a flexibility that is important to the interaction with the microbial targets, which can contribute to their antimicrobial activity [19]. In this study, the CD analysis demonstrated the structural variability of StigA25 and StigA31 in different solvents, showing a high percentage of α-helix in hydrophobic environments similar to anionic membranes and a predominant random coil conformation when submitted to polar solvents. This structural variability was already observed for the native peptide [14]. Furthermore, Stigmurin, StigA25, and StigA31 demonstrated structural stability in a broad temperature and pH range when analyzed by CD in the presence of SDS 20 mM, returning to its initial conformation after cooling.

StigA25 and StigA31 demonstrated broad-spectrum antimicrobial activity, which was higher than that presented by Stigmurin. The native peptide demonstrated activity against Gram-positive bacteria, but was not effective against Gram-negative bacteria at the higher tested dose (150 µM). Furthermore, the analog peptide activity was higher than that of standard antibiotics Vancomycin, Gentamicin, and Amphotericin B for the most tested strains after 24 h incubation, showing that the analogs generated in this study are promising candidates for the development of anti-infective therapeutic agents. The higher antimicrobial activity in vitro of StigA31 (+7) when compared to Stigmurin (+2) and StigA25 (+5) was also demonstrated in the theoretical analysis obtained by molecular dynamics. In the secondary structure analysis by CD we did not observe the increment in the helical conformation of Stig25 and Stig31 in comparison with Stigmurin, with the positive net charge increment being the main factor related to the action spectrum and antimicrobial activity increase.

Previous studies suggest that AMPs can interact with high affinity with lipopolysaccharide (LPS), a glycolipid present in the Gram-negative bacteria outer membrane [22]. The potent activity of the analog peptides StigA25 and StigA31 against bacteria strains can be associated with their cationicity, compared to Stigmurin. The peptide–bacteria interaction in a complex and possibly specific process, being the product of the more efficient interaction of the peptide with some microorganism compounds [23]. LPSs provide a protective barrier against molecules higher than 1000 Da, then acting as a defensive mechanism against antibiotics and AMPs [24], which suggests that the peptides reported here do not act through this mechanism, since these AMPs are large for crossing the microbial pores and reach the cell membrane. The antimicrobial peptides, due to their cationic and amphiphilic nature, may accumulate on the bacteria surface, leading to cell wall rupture through the interaction with their compounds [25]. A similar action mechanism was described for Kn2-7, an analog peptide from the native peptide BmKn2 isolated from the scorpion *Mesobuthus martensii* Karsch, which promoted cell wall rupture of *S. aureus* and *E. coli* through the LTA and LPS binding, respectively [26]. SEM data indicate that the mechanism of death of peptides is distinct from that of most cationic peptides, which generally influence membrane permeability or even form pores on the bacterial surface, seriously disrupting cell morphology [1]. Our study therefore provides evidence that Stigmurin and its analogues may induce bacterial suppression by disrupting the cell wall without promoting cell lysis. In an earlier study with Bac2A peptide analogs, drastic changes were also observed in the cell surface of *S. aureus*, with protuberances and cracks in the cell wall, not demonstrating interference in the inner membrane of the cells. In addition, the Bac2A peptide kills *S. aureus* without promoting cell lysis [27].

Regarding the antiparasitic activity, the three tested peptides inhibited the epimastigote forms of the *T. cruzi* Y strain. No significant difference was observed between the inhibition caused by the analog peptides; though when compared with the native peptide Stigmurin in an earlier study, both analog peptides showed higher antiparasitic activity [15]. Various antimicrobial peptides from aquatic animals have been isolated and tested against epimatigote forms of *T. cruzi*; however, they did not show antiparasitic activity for these strains [28], different from the analog peptides StigA25 and StigA31, which demonstrated a high inhibition rate. Similar activity was also shown for other analog peptides from the scorpion peptide Stigmurin [15] and various analog peptides from scorpion venom with significant activity against trypanosomatides [29]. When tested in trypomastigote forms of the *T. cruzi* and Y strains we observed the same pattern for epimastigote, with the analogs peptides showing higher activity than the native peptide Stigmurin. When comparing the analog peptides with benznidazol (where the inhibition rate for the same Y strain was calculated in an earlier study), at the same concentration and exposure time, both analog peptides presented higher antiparasitic activity in the lower concentrations tested [15]. Benznidazol is the main drug used in the treatment of Chaga’s disease, showing efficacy after 72 h incubation in tripomastigote forms of the *T. cruzi* Y strain [30]. Therefore, the analog peptides were more effective in lower incubation time with lower concentrations. Our results demonstrate that the analog peptides generated are potential candidates for additional investigation and application as therapeutic agents for Chagas disease, as an alternative or in addition to the conventional treatment, considering their antiparasitic properties. 

AMPs can interact with the erythrocyte surface through the interaction with sialic acid presented in the glycoproteins or glycosphingolipids, which constitute the glycocalyx [3]. La Salude Bea and collaborators [31] studied analogs from the native peptide BmKn1 found in the scorpion *Buthus martensii* Karsch venom. They suggested that modification of the native AMP sequence, which results in the increase of positive net charge through the addition of lysine residues, leads to the increase in the antimicrobial and hemolytic activity. Under the conditions that we evaluated here, the analog peptides presented low hemolysis rate at the concentrations at which they presented antimicrobial activity, demonstrating potential therapeutic application of these bioactive peptides.

## 4. Materials and Methods

### 4.1. Peptide Synthesis

**Figure A1:**
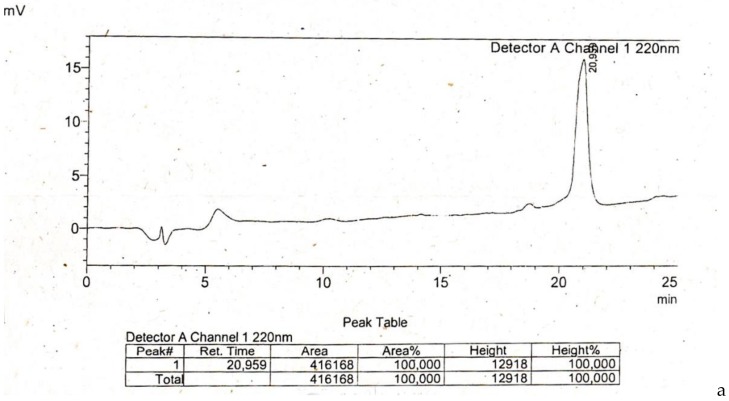
Analysis by high-performance liquid chromatography (HPLC) of Stigmurin peptide.

**Figure A2:**
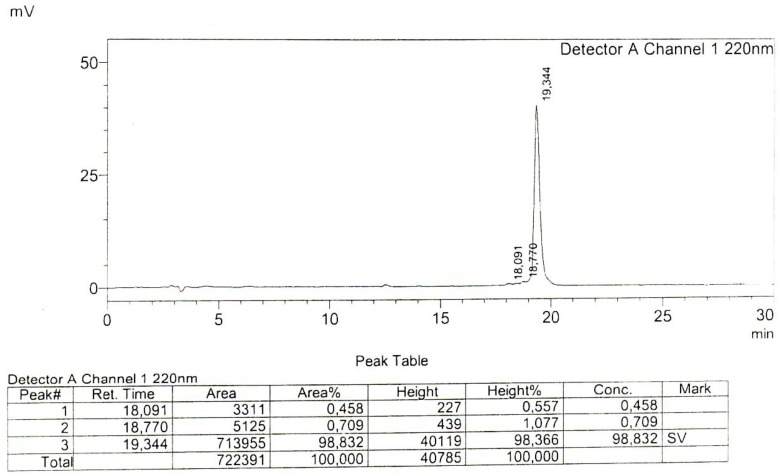
Analysis by high-performance liquid chromatography (HPLC) of StigA25 peptide.

**Figure A3:**
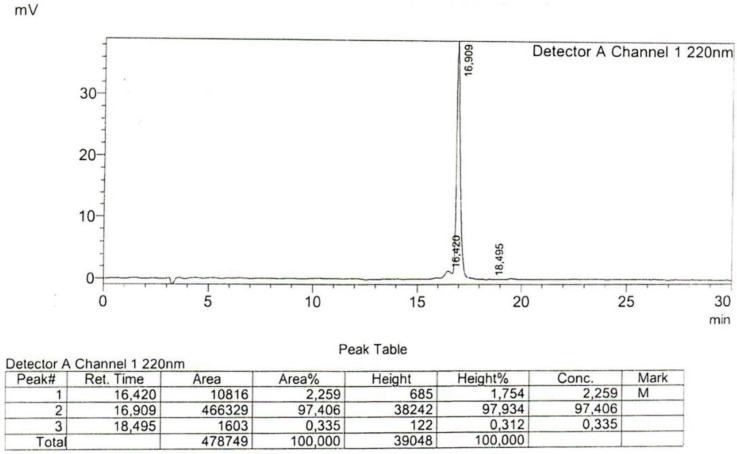
Analysis by high-performance liquid chromatography (HPLC) of StigA31 peptide.

**Figure A4:**
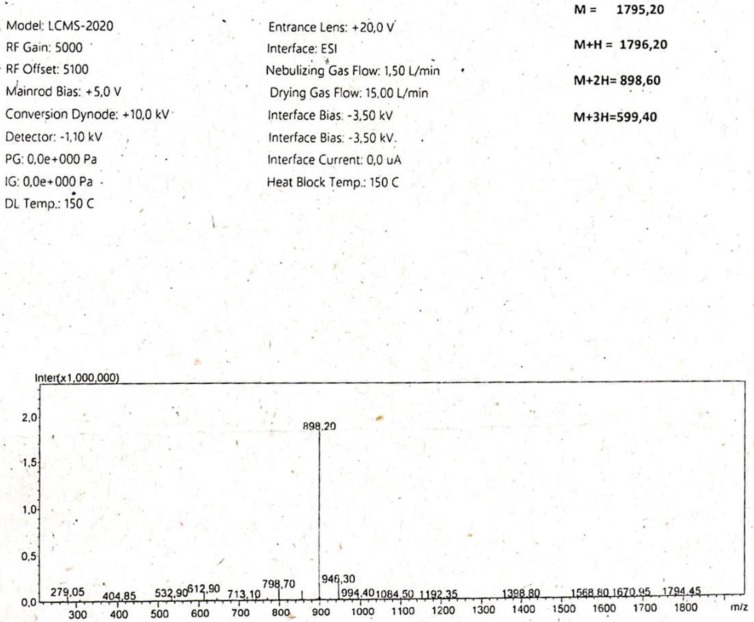
Mass spectroscopy analysis of peptide Stigmurin.

**Figure A5:**
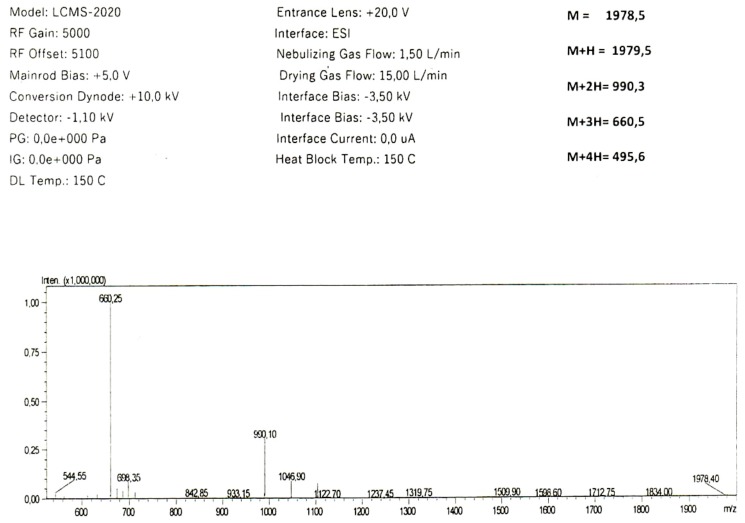
Mass spectroscopy analysis of peptide StigA25.

**Figure A6:**
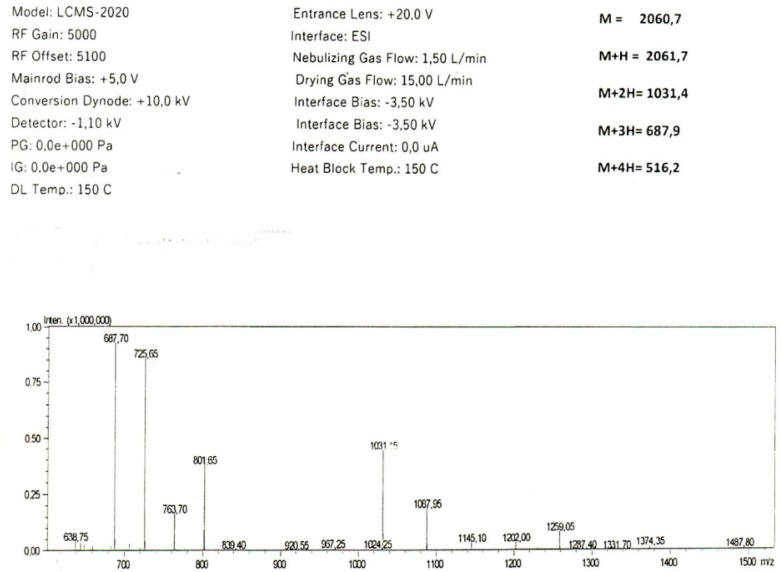
Mass spectroscopy analysis of peptide StigA31.

The lyophilized peptide Stigmurin (FFSLIPSLVGGLISAFK-NH_2_) and the analogs StigA25 (FFSLIPSLVKKLIKAFK-NH_2_) and StigA31 (FFKLIPKLVKKLIKAFK-NH_2_) were obtained commercially with C-terminal amidation from *AminoTech Pesquisa e Desenvolvimento* (Minas Gerais, Brazil). The peptides were stored at −20 °C until use. The purity (≥98%) was confirmed by high performance liquid chromatography and the molecular weight confirmed by mass spectrometry (Figure A1, Figure A2, Figure A3, Figure A4, Figure A5 and Figure A6) in the Appendix A.

### 4.2. In Silico Analysis

Stigmurin and the analog peptide physicochemical properties (hydrophobicity, hydrophobic moment and net charge) were predicted using HeliQuest server (http://heliquest.ipmc.cnrs.fr/) and the theoretical secondary structural conformation, using PORTER server (http://distill.ucd.ie/porter/).

### 4.3. Molecular Modeling

The peptide tridimensional structure prediction was obtained by the ab initio method, using AIDA software (http://ffas.burnham.org/AIDA/). The theoretical models were validated using the Molprobity server (http://molprobity.biochem.duke.edu/) [32] and analyzed parameters, such as the Ramachandran plot, bond sizes, and angles, to optimize the obtained theoretical structure. The structures were visualized using UCSF Chimera software (version 1.8.1).

### 4.4. Molecular Dynamics Simulations (MD)

**Figure A7:**
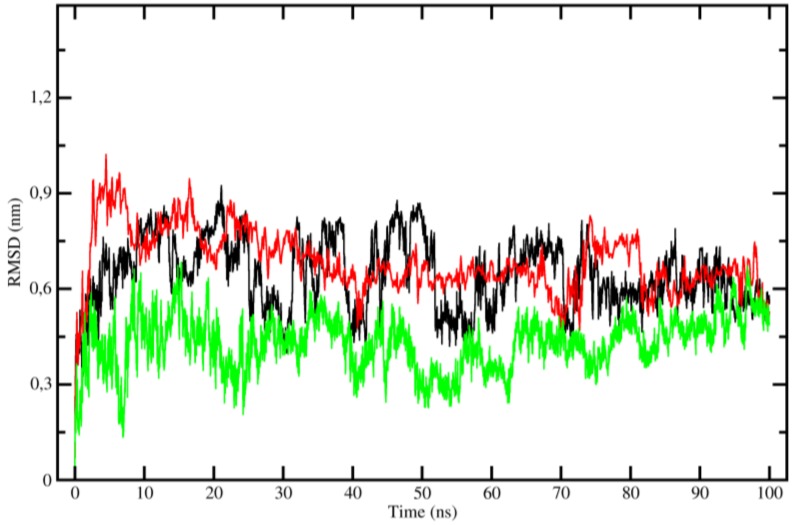
RMSD profile for C-α atoms over 100 ns from MD simulations. Stigmurin (black line), StigA25 (red line), and StigA31 (green line).

The lipid bilayer model were constructed for the theoretical study with the purpose of simulating the Gram negative membranes from bacteria. To construct this model, a mixture with a 1:3 molar ratio of anionic 1-palmitoyl-2-oleoyl-sn-glycero-3-phosphoglycerol (POPG) to 1-palmitoyl-2-oleoyl-sn-glycero-3-phosphoethanolamine (POPE) was used [16]. The Membrane Builder module of the CHARMM-GUI [33] server was used for the construction of lipid bilayers. The initial structures of peptides generated through MolProbity were simulated separately in a system containing only water molecules for 100 ns. (RMSD, Figure A7). The final structures from these simulations were placed in the upper region of the membranes at approximately 2.0 nm above the lipid bilayer surface, with the initial position being the same for all peptides. This initial distance between peptides and lipids was also used on recent publications since it allows the free movement of peptide and your possible interaction with membrane [16,34,35]. The lipid bilayer used for all membrane simulations was positioned in the xy plane of the box and was composed of 120 lipids between POPE and POPG molecules. A ~40 Å thick layer of water (z-axis) was always maintained on both sides of the lipid bilayer. All MD simulations were performed using the Gromacs 5.1.4 [36] package implemented with the CHARMM36 force field [37]. TIP3P [38] water molecules were used to solvate the simulated systems. The systems neutralization was achieved through the addition of counter ions. The Leap-Frog [39] algorithm was applied to integrate the motion equation with time step of 2.0 fs. The long-range interactions were modeled using particle-mesh Ewald sum, PME [40], with a cutoff of 1.2 nm. The van der Waals interactions were also calculated using the same threshold. Bonds involving hydrogen atoms were restrained using LINCS [41]. The Nosé–Hoover thermostat [42] was used to fix the system temperature (310 K) in all production simulations, while the system pressure was controlled using a Parrinello–Rahman barostat in the NPT simulations [43]. The geometry of the systems was minimized by the steepest descent algorithm for 5000 steps with tolerance of 500 kJ mol^−1^ nm^−1^. Two short 25-ps equilibrium dynamics tests were performed with NVT ensemble, followed by four short 25 ps, 100 ps, 100 ps, and 225 ps equilibrium dynamics with NPT ensemble. Finally, 100 ns production MD simulation using NVT ensemble was performed for each system in order to determine the peptide (Stigmurin, StigA25 or StigA31) adsorption with lipid bilayers. A system containing only the lipid bilayer without the presence of the peptides was also evaluated.

To analyze the adsorption between peptides and lipid bilayer we used the Interaction Potential Energy (IPE), which can be defined as the total interaction energy between two groups, (the sum of electrostatic and van der Waals contributions), and it was computed according to the equation:

(1) $${\mathrm{IPE}}_{i,j}=\sum_{i}^{N_{i}}\sum_{j\neq i}^{N_{j}}V_{\mathrm{vdW}}\left(r_{ij}\right)+V_{\mathrm{Ele}}\left(r_{ij}\right),$$

where *IPE_i,j_* is the interaction energy between a group of atoms *i* and a group of atoms *j*, and N_i_ and N_j_ are the total number of atoms on groups *i* and *j*, $V_{\mathrm{Ele}}$ and $V_{\mathrm{vdW}}$ are the terms corresponding to electrostatic and van der Waals contribution, respectively. This parameter is often used to evaluate interaction energies in protein–ligand and protein–protein systems [44], but can also be applied to quantify interaction between specific amino acids and the surrounding molecules [45]. For IPE calculations, all peptide atoms were considered in the calculation. It was separated by amino acid for the results in Figure 4. For the lipid bilayer group, only the hydrophilic head atoms of POPE and POPG were considered.

### 4.5. Circular Dichroism Structural Characterization

Stigmurin, StigA25, and StigA31 secondary structure characterization was performed by circular dichroism, using a spectropolarimeter JASCO-810 at pH 7.0 and 25 °C using a Peltier system for temperature control. Spectra were analyzed in water, PBS 10 mM, sodium dodecyl sulfate 20 mM and 40 mM and trifluoroethanol (TFE) in a concentration range of 20% to 70% (*v*/*v*); the peptides were 0.3 mg/mL final concentration. The analyses were performed at a wavelength range of 190–260 nm, and detected 10 times at 50 nm·min^−1^ velocity. Results were obtained at millidegrees (1/1000°) and converted to molar ellipticity [*θ*] using the expression [46]:

(2) $$\frac{[\theta ]\;=\;\theta \;\times \;100\;\times \;M}{C\;\times \;I\;\times \;Nr}$$

where *θ* corresponds to the ellipticity in millidegrees, *I* is the optical path (cm), *C* corresponds to the peptide concentration (mg/mL), *M* is the molecular mass, and *Nr* corresponds to the number of amino acid residues. The CD spectra deconvolution was obtained using the Dichroweb server [47] and CDSSTR algorithm [48]. The spectra base line was corrected by subtracting the solvents in identical conditions from the spectra.

### 4.6. Structural Stability by Circular Dichroism

The peptide stability was evaluated in SDS 20mM in the pH range of 3.0 to 9.0 and temperature from 2 to 98 °C. Results were obtained as described in Section 4.5.

### 4.7. Hemolytic Activity

Peptide toxicity was evaluated by hemolytic assay following the methods described by Menezes and collaborators [49] with modifications. The erythrocyte suspension (2% *v*/*v*) was washed three times in saline 0.9% (pH 7.4) and centrifuged at 200× *g* for 10 min. The red blood cells were incubated for 60 min at 37 °C with Stigmurin, StigA25, or StigA31 in different concentrations (1.2–150 μM) and centrifuged at 200× *g* for 10 min. The hemoglobin presented in the supernatant rate was quantified at 540 nm in a microplate reader (Epoch-Biotek, Winooski, VT, USA), indicating the erythrocytes lysis. Triton X-100 1% (*v*/*v*) was used as positive control (100% cell lysis) and saline 0.9% as negative control (0% cell lysis).

### 4.8. Microorganisms

Stigmurin, StigA25, and StigA31 antibacterial activity was evaluated against Gram-positive bacteria including *Staphylococcus aureus* (ATCC 29213), *Staphylococcus epidermidis* (ATCC 12228), and *Enterococcus faecalis* (ATCC 4028), and Gram-negative bacteria including *Escherichia coli* (ATCC 25922), *Pseudomonas aeruginosa* (ATCC 27853), and *Enterobacter cloacae* (ATCC 13047). The antifungal activity was evaluated against yeasts: *Candida glabatra* (ATCC 90030), *Candida albicans* (ATCC 90028), and *Candida krusei* (ATCC 6258). All tested strains were obtained from the Clinical Microbiology Laboratory at the Clinical and Toxicological Analysis Department of the University Federal of Rio Grande do Norte. The microorganisms were conserved in nutrient agar (HIMEDIA^®^, Mumbai, India) at 4 °C.

### 4.9. In Vitro Antimicrobial Activity

The antimicrobial activity analysis was performed using the broth microdilution [50,51] with modifications. In a 96-well plate, a microorganism suspension with 10^6^ CFU/mL (bacteria) or 10^5^ CFU/mL (yeasts) in Mueller Hinton (MH) broth were added to different concentrations of Stigmurin, StigA25, or StigA31 (1.2–150 μM) or saline solution 0.9% and incubated at 35 ± 2 °C, at 200 rpm for 24 h. After incubation, the optical density was evaluated at 595 nm in a microplate reader (Epoch Biotek). Wells containing broth and saline solution 0.9%, without microorganisms, were used as a sterility control (negative growth control), while wells containing broth, microorganisms, and saline solution 0.9% were the positive growth control. For the microbial sensibility profile control, Vancomycin (64–0.5 µg/mL) was added to the Gram-positive bacteria, Gentamicin (128–1.0 µg/mL) to the Gram-negative bacteria, and Amphotericin B (32–0.3 µg/mL) to yeasts. The minimum inhibitory concentration was determined as the minimum required peptide concentration in which the 595 nm absorbance was identical to the negative control. All assays were performed in quadruplicate.

### 4.10. In Vitro Antiparasitic Activity

The antiparasitic activity was evaluated in the epimastigote and trypomastigote forms of *Trypanosoma cruzi* Y strain following the methodology described [52]. The epimastigote parasites were incubated for 11 days at 27 ± 2 °C in Liver Infusion Triptose medium (LIT) until the stationary phase and diluted to 1 × 10^7^ parasites/mL. In a 96-well plate the parasite solution was added to the peptides in serial dilution (25–1.6 μM), and incubated for 12 and 24 h at 27 ± 2 °C. Afterwards, 3-(4,5-dimetiltiazol-2-ilo)-2,5-difeniltetrazol (MTT) at 5 mg/mL was added and then the plate was incubated for 75 min, when finally the solubilization solution (HCL 0,01 N + SDS 10%) was added and the plate was incubated for 30 min and then read on the microplate reader at 595 nm. For the anti-trypomastigote assays, the epimastigote forms were transformed into trypomastigotes by adding 1 mL of the epimastigote culture solution to 9 mL of LIT medium, and incubated for 25 days at 27 ± 2 °C. The experiments were performed following the methodology described for the epimastigote form.

### 4.11. Scanning Electron Microscopy

The morphology of *S. aureus* cells incubated with Stigmurin, StigA25 and StigA31 (2× MIC, 1× MIC and ½× MIC) for 18h was visualized by SEM (SEM-FEG ZEISS AURIGA 40). In general, 1× 10^8^ mid-logarithmic-phase *S. aureus* cells were treated with peptide, and a no-peptide control was included. After incubation, bacteria were pelleted by centrifugation at 3000 rpm for 10 min, followed by washing twice with 1M PBS. Cells were then fixed with a 2.5% glutaraldehyde–PBS solution at 4 °C for 4 h. For SEM, the bacteria were further treated with 20%, 50%, 80%, and 100% ethyl alcohol for 15 min and centrifuged at 13,000 rpm for 10 min for dehydration. The bacterial pellet was resuspended in 100% ethyl alcohol and air-dried. The prepared bacterial samples were sent to the Structural Characterization Laboratory of Materials of Federal University of Rio Grande do Norte (Natal, Brazil) for imaging.

### 4.12. Statistical Analysis

All assays were performed at least three times (for antiparasitic activity and hemolytic activity). The data are expressed as mean ± standard deviation. The statistical analysis was performed using one-way analysis of variance (ANOVA) followed by Tukey’s test, with GraphPad Prism software (version 5.00, GraphPad, San Diego, CA, USA). Data were considered significant when the *p* value was less than 0.05 (*p* < 0.05).

## 5. Conclusions

The analog peptides generated in this study exhibited predominant α–helix conformation in hydrophobic environments, presenting high stability over a broad temperature and pH range, similar to the native peptide Stigmurin. The substitutions in the native sequence for lysine residues resulted in hydrophobicity and positive net charge increase, concomitantly with an improvement in the antimicrobial properties of the analog peptides, which have a higher activity and a broader spectrum when compared to Stigmurin. At the MIC, the analog peptides presented a low hemolysis rate. Antiparasitic activity was also demonstrated for the analogs, with a high inhibition rate for the *T. cruzi* strains. The molecular dynamics results showed that the peptide StigA31, mainly mutated residues, presented the highest propensity of interaction with the microbial membranes among all evaluated peptides, corroborating the obtained in vitro data, where this peptide showed higher antimicrobial activity against the tested strains when compared with the native peptide and StigA25. Therefore, this study shows the potential therapeutic application of the analog peptides from scorpion venom as anti-infective agents.

## Figures and Tables

**Table 1:** Physiochemical properties and secondary structure predictions in silico of the peptides Stigmurin, StigA25, and StigA31.

Peptides	Sequences ^a^	Secondary Structure ^b^	α-Helix	H	µH	Charge
Stigmurin	FFSLIPSLVGGLISAFK-NH_2_	CCCCC**HHHHHHHHHHH**C	64.7 %	0.89	0.57	+2
StigA25	FFSLIPSLV**KK**LI**K**AFK-NH_2_	CCC**HHHHHHHHHHH**CC	70.5 %	0.73	0.70	+5
StigA31	FF**K**LIP**K**LV**KK**LI**K**AFK-NH_2_	C**HHHHHHHHHHHHHHH**C	88.2 %	0.61	0.80	+7

^a^ Characters in bold indicate the modifications. H: Hydrophobicity (calculated using Heliquest); µH: hydrophobic moment; ^b^ H: α-helix conformation; and C: Random structure.

**Table 2:** Secondary structure percentage of Stigmurin, StigA25, and StigA31 obtained by CD.

Solvent	α-Helical %			β-Sheet %			Random Coil %		
	Stig *	StigA25	StigA31	Stig *	StigA25	StigA31	Stig *	StigA25	StigA31
Water	3	2	2	17	25	17	79	72	79
PBS 10 mM	3	2	2	13	28	19	82	68	78
SDS 20 mM	68	41	54	4	18	9	27	41	36
SDS 4 mM **	65	42	56	6	19	15	28	40	28
TFE 20%	58	40	49	10	17	16	32	42	34
TFE 30%	68	41	52	7	19	14	24	40	35
TFE 40%	70	40	49	6	18	17	23	41	33
TFE 50%	65	41	50	6	21	15	28	39	33
TFE 60%	59	43	49	12	17	15	29	40	35
TFE 70%	59	39	46	11	19	16	29	41	17

Secondary structures expressed as percentage and obtained by CDSSTR algorithm. * Peptide in its native composition. ** Below critical micelle concentration—CMC.

**Table 3:** Percentage of the secondary structure obtained by CD at different pH.

Potential of Hydrogen (pH)	α-Helical %			β-Sheet %			Random Coil %		
	Stig *	StigA25	StigA31	Stig *	StigA25	StigA31	Stig *	StigA25	StigA31
**3**	70	50	53	5	12	11	24	37	36
**4**	69	49	54	6	13	12	25	37	33
**5**	69	58	59	5	16	12	26	27	29
**6**	76	48	52	10	14	13	15	39	35
**7**	69	49	54	5	14	13	26	36	34
**7.4**	79	47	53	5	16	12	16	36	35
**8**	69	49	51	6	13	12	24	37	36
**9**	80	51	57	5	12	12	14	36	30

Secondary structures expressed as percentage and obtained by CDSSTR algorithm. * Peptide in its native composition.

**Table 4:** Determination of minimum inhibitory concentrations (MICs) of Stigmurin, StigA25, and StigA31.

Strains	Minimum Inhibitory Concentration (MIC)			
	Stigmurin (µM)	StigA25 (µM)	StigA31 (µM)	Antibiotics (µM) *
**Gram-positive**				
*Staphylococcus aureus* (ATCC 29213)	9.4	1.2	2.3	5.5
*Staphylococcus epidermidis* (ATCC 12228)	9.4	2.3	2.3	22.1
*Enterococcus faecalis* (ATCC 4028)	>150	4.7	1.2	5.5
**Gram-negative**				
*Pseudomonas aeruginosa* (ATCC 27853)	>150	4.7	2.3	8.4
*Escherichia coli* (ATCC 25922)	>150	2.3	1.2	4.2
*Enterobacter cloacae* (ATCC 13047)	>150	18.8	4.7	67
**Yeasts**				
*Candida albicans* (ATCC 90028)	37.5	9.4	4.7	34
*Candida glabrata* (ATCC 90030)	>150	9.4	4.7	3.2
*Candida krusei* (ATCC 6258)	>150	9.4	4.7	34

* The standard antibiotics used were Vancomycin (for Gram-positive strains), Gentamicin (for Gram-negative strains), and Amphotericin B (for yeasts).
